# Inhibition of Mixed Biofilms of *Candida albicans* and *Staphylococcus aureus* by β-Caryophyllene-Gold Nanoparticles

**DOI:** 10.3390/antibiotics12040726

**Published:** 2023-04-07

**Authors:** Fazlurrahman Khan, Nazia Tabassum, Geum-Jae Jeong, Won-Kyo Jung, Young-Mog Kim

**Affiliations:** 1 Marine Integrated Biomedical Technology Center, The National Key Research Institutes in Universities, Pukyong National University, Busan 48513, Republic of Korea; 2 Research Center for Marine Integrated Bionics Technology, Pukyong National University, Busan 48513, Republic of Korea; 3 Department of Food Science and Technology, Pukyong National University, Busan 48513, Republic of Korea; 4 Major of Biomedical Engineering, Division of Smart Healthcare and New-Senior Healthcare Innovation Center (BK21 Plus), Pukyong National University, Busan 48513, Republic of Korea

**Keywords:** mixed biofilms, β-caryophyllene, gold nanoparticles, *Candida albicans*, *Staphylococcus aureus*

## Abstract

Polymicrobial biofilms, consisting of fungal and bacterial pathogens, often contribute to the failure of antimicrobial treatment. The growing resistance of pathogenic polymicrobial biofilms to antibiotics has led to the development of alternative strategies to combat polymicrobial diseases. To this end, nanoparticles synthesized using natural molecules have received significant attention for disease treatment. Here, gold nanoparticles (AuNPs) were synthesized using β-caryophyllene, a bioactive compound isolated from various plant species. The shape, size, and zeta potential of the synthesized β-c-AuNPs were found to be non-spherical, 17.6 ± 1.2 nm, and -31.76 ± 0.73 mV, respectively. A mixed biofilm of *Candida albicans* and *Staphylococcus aureus* was used to test the efficacy of the synthesized β-c-AuNPs. The results revealed a concentration-dependent inhibition of the initial stages of formation of single-species as well as mixed biofilms. Furthermore, β-c-AuNPs also eliminated mature biofilms. Therefore, using β-c-AuNPs to inhibit biofilm and eradicate bacterial-fungal mixed biofilms represents a promising therapeutic approach for controlling polymicrobial infections.

## 1. Introduction

Polymicrobial interactions at the interspecies or cross-kingdom level significantly influence pathogenic behaviors, including virulence, growth, host immune system tolerance, and antimicrobial resistance [1,2,3]. The interactions between *Staphylococcus aureus* and *Candida albicans* are major contributors to skin, mucosal, and bloodstream infections [4,5,6]. *S. aureus* is the third most common bacterial pathogen that is often responsible for various infections such as blood, burn wound, and catheter infections, as well as denture stomatitis and peri-implantitis [5,7,8]. The effect of injecting sublethal doses of *S. aureus* and *C. albicans* into animal models on mortality rates have been previously investigated [9,10]. Pathogenicity due to the synergistic effect of *S. aureus* and *C. albicans* also increases antimicrobial resistance. Physical adherence and chemical signaling have been identified to be responsible for the synergistic interactions between *S. aureus* and *C. albicans*. *S. aureus* physically adheres onto the surface of *C. albicans*, which is coated with an extracellular matrix [11,12]. The involvement of secretory molecules produced by *S. aureus* and *C. albicans* in interspecies interactions further demonstrated the presence of chemical interactions. Thus, biofilm formation is enhanced, and drug resistance is increased [12].

The World Health Organization (WHO) has designated *S. aureus* and *C. albicans* as priority microbial pathogens, which further emphasizes the need for drug discovery against these pathogens [13,14]. The aim of this study was to synthesize nanoparticles using natural bioactive compounds to enable the development of alternative treatment strategies against *S. aureus* and *C. albicans.* Nanoparticles synthesized using natural compounds have several advantages, including biocompatibility, high abundance, low cytotoxicity, and environment friendliness [15]. Such green nanoparticles are also particularly advantageous as they are stable, biocompatible, and easy to synthesize, with low costs and without toxic chemical waste generation. [15,16,17]. Metal ions, such as gold and silver, can be reduced using agents found in various organisms to produce stable metal or metal oxide nanoparticles [18,19]. In this regard, gold and silver nanoparticles are particularly interesting due to their antibacterial activity via attachment to the cell membrane surface, penetration of the bacterial cell, and the release of Ag^+^ and Au^+^ ions [20,21].

The volatile sesquiterpene β-caryophyllene (C_15_H_24_) has been reported in several plant species such as *Psidium guajava*, *Arabidopsis thaliana*, *Eugenia caryophyllata*, *Artemisia annua* L., *Aquilaria crassna*, *Ocimum sanctum*, *Syzygium cumini*, and *Spiranthera odoratissima* [22,23,24]. Due to its low toxicity and high biocompatibility, β-caryophyllene is commonly used as a flavoring agent in the food industry. β-caryophyllene also exhibits a variety of other biological activities, such as antimicrobial, anti-inflammatory, anxiolytic, anticancer, antioxidant, and anti-spasmodic activities [24].

The antibacterial and antibiofilm effects of nanoparticles produced using β-caryophyllene have been investigated in several studies previously (Table 1). However, previous studies on this compound have largely focused on single-species biofilms or mono-planktonic cultures [25,26,27]. Here, we synthesized gold nanoparticles using β-caryophyllene (β-c-AuNPs) as the reducing agent. Being a terpene, β-caryophyllene has been previously shown to display bioreduction and metal ion stabilization into the zerovalent state as a nanoparticle [28].

The synthesized β-c-AuNPs were fully characterized using several instrumental techniques. Moreover, their antimicrobial activities against *S. aureus* and *C. albicans* were investigated. Since the majority of previously developed nanoparticles have been evaluated against single-species biofilms [39,40,41], the inhibitory activity of β-c-AuNPs on polymicrobial biofilms is a novel contribution to efforts in combatting the emergence of antimicrobial resistance.

## 2. Results

### 2.1. Synthesis and Characterization of β-c-AuNPs

**Figure 1:**
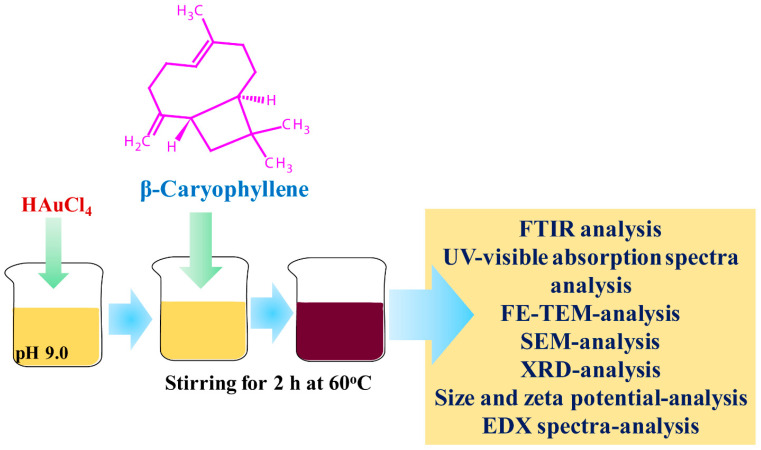
Steps employed in the synthesis and characterization of β-c-AuNPs.

Figure 1 shows the procedure of the synthesis of β-c-AuNPs followed in this study. A color change from yellow to deep wine-red was used to confirm the formation of AuNPs.

**Figure 2:**
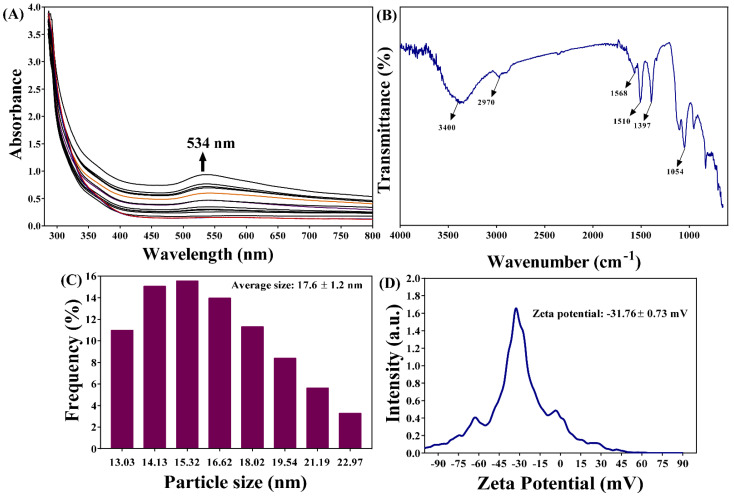
(**A**) Absorption spectra of β-c-AuNPs, (**B**) FTIR spectra, (**C**) size distribution, and (**D**) zeta potential.

The UV-vis absorption spectra showed a continuous increase, with a maximum peak at 534 nm (Figure 2A). An FTIR analysis revealed that β-c-AuNPs exhibit vibration band characteristics at 3400, 2970, 1568, 1510, 1397, and 1054 cm^−1^, respectively (Figure 2B). The broadband at 3400 cm^−1^ and a shoulder at 2970 cm^−1^ represent the hydroxyl group of the compound, which is involved in metal ion reduction [28]. The peak at 1397 cm^−1^ corresponds to the O-H bending of phenol, whereas the peak at 1054 cm^−1^ corresponds to the CO-O-CO stretching of anhydride. The average size of β-c-AuNPs was determined to be 17.6 ± 1.2 nm (Figure 2C) using dynamic light scattering. The zeta potential of β-c-AuNPs was measured as −31.76 ± 0.73 mV (Figure 2D).

**Figure 3:**
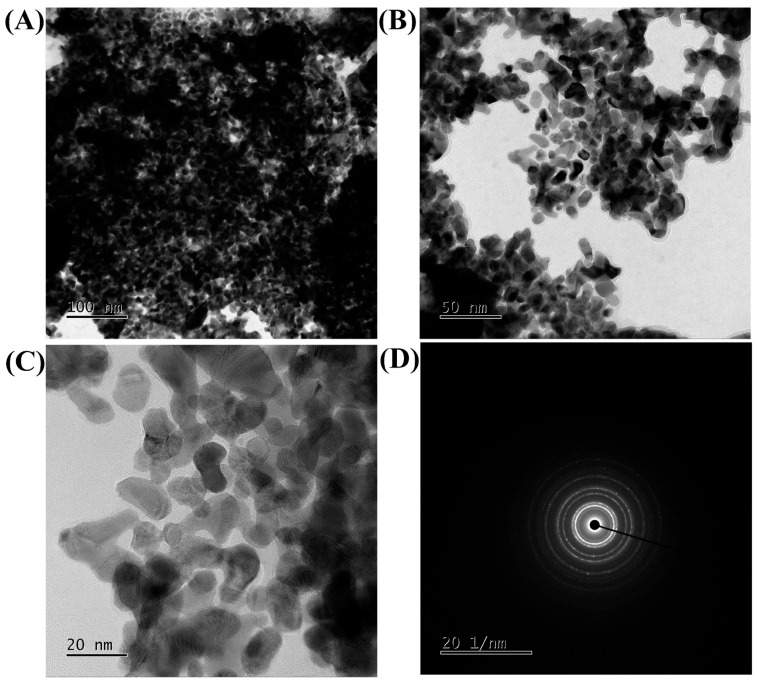
FE-TEM images of β-c-AuNPs at 100 nm (**A**), 50 nm (**B**), 20 nm (**C**), and SAED (**D**).

**Figure 4:**
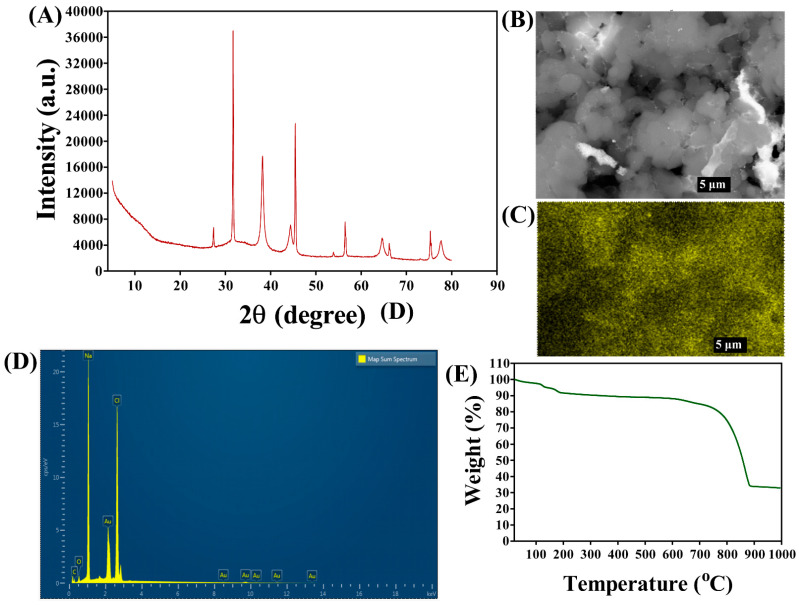
(**A**) XRD analysis of β-c-AuNPs, (**B**) SEM imaging, (**C**) mapping of the Au element in β-c-AuNPs, (**D**) EDX spectra, and (**E**) TGA analysis.

The field emission transmission electron microscope (FE-TEM) analysis of β-c-AuNPs morphology revealed a non-uniform shape (Figure 3A–C). The scanning electron microscopy (SEM) analysis further confirmed the non-uniform shape of β-c-AuNPs as well (Figure 4B). The presence of Debye–Scherrer rings in selected area diffraction pattern (SAED) confirmed the crystalline nature of β-c-AuNPs (Figure 3D). Furthermore, an X-ray diffractometer (XRD) analysis revealed distinctive peaks at *2ϴ* values of 38.2°, 45.5°, 55.4°, 66.2°, 75.3°, and 77.5°, which are similar to the characteristic peaks observed in other AuNPs obtained using green synthesis methods (Figure 4A). The presence of Au in β-c-AuNPs was also confirmed by elemental mapping (Figure 4C). The presence of a gold signal in β-c-AuNPs was confirmed by energy dispersive X-ray (EDX) analysis (Figure 4D). Thermal gravimetric analysis (TGA) of the synthesized AuNPs confirmed a steady weight loss from 20 to 600 °C, with a total loss of up to 900 °C. (Figure 4E).

### 2.2. Determination of the Minimum Inhibitory Concentration of β-c-AuNPs 

**Figure 5:**
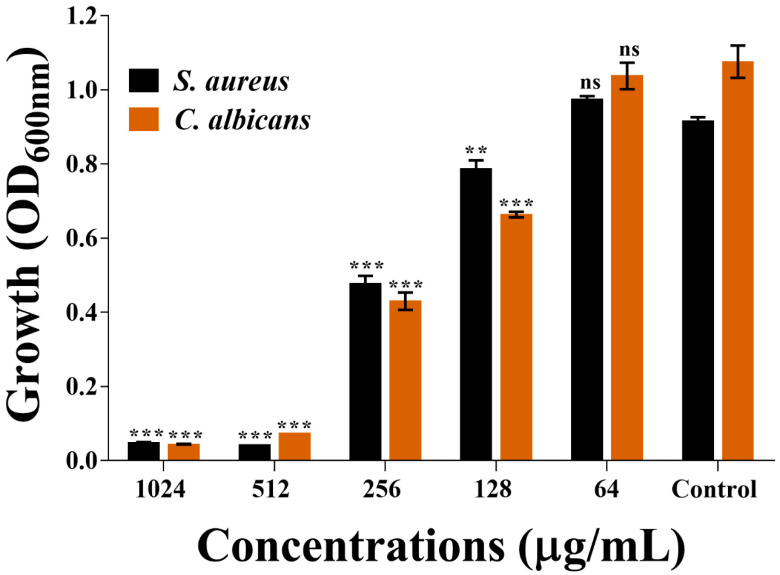
Growth study for determining the MIC values of β-c-AuNPs against *S. aureus* and *C. albicans*. *** *p* < 0.0001 and ** *p* < 0.01 are statistically significant compared to the control, whereas ns is non-significant.

The antimicrobial activities of β-c-AuNPs were investigated using the microbroth dilution method. The minimum inhibitory concentration (MIC) of β-c-AuNPs against *S. aureus* and *C. albicans* (Figure 5) was found to be 512 μg/mL. We also determined the MIC values of β-caryophyllene for these pathogens, and found that the values were different from those of β-c-AuNPs. The MIC of β-caryophyllene against *S. aureus* and *C. albicans* was found to be >2048 μg/mL, which is four-fold higher than the MIC of β-c-AuNPs. The MIC values of tetracycline and fluconazole as control drugs against *S. aureus* and *C. albicans* were found to be >0.5 μg/mL and >32 μg/mL, respectively. These values are consistent with previously published values [42].

### 2.3. Inhibitory Activity of β-c-AuNPs on Biofilms

**Figure 6:**
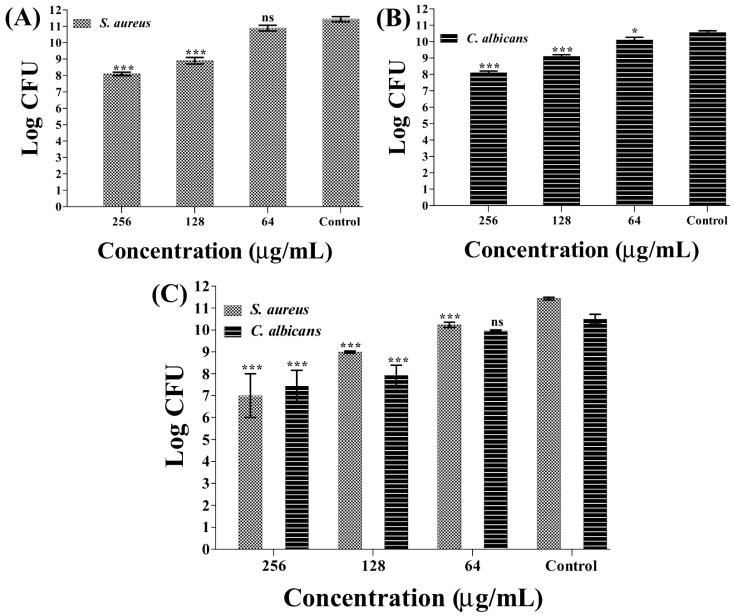
Inhibition of the initial stage of biofilm formation by β-c-AuNPs. (**A**) CFU of *S. aureus* cells, (**B**) CFU of *C. albicans* cells, and (**C**) CFU of *C. albicans* and *S. aureus* cells from mixed biofilms. *** *p* < 0.0001 and * *p* < 0.05 are statistically significant compared to the control, whereas ns is non-significant.

The counting of microbial colony forming units (CFU) was used to examine the effects of sub-MIC β-c-AuNPs on the initial-stage biofilms of *S. aureus* and *C. albicans*, as well as the mixed biofilm. The biofilm inhibitory effects of β-c-AuNPs on *S. aureus* were found to be concentration-dependent (Figure 6A). The maximum level of inhibition on the *S. aureus* biofilm was observed at a concentration of 256 μg/mL, with a reduction of 3.34 log CFU compared to the control. β-c-AuNPs were found to inhibit *C. albicans* biofilms in a concentration-dependent manner as well. Here, the maximum level of inhibition was found to be a 2.45 log CFU reduction of cells at a concentration of 256 μg/mL of β-c-AuNPs (Figure 6B). The inhibition of the initial stage of the mixed biofilm of *S. aureus* and *C. albicans* was also found to be concentration-dependent, as measured by the CFU values of *S. aureus* and *C. albicans* on a selective agar plate containing an antibiotic or antifungal agent (Figure 6C). The reduction in the log CFU value of *S. aureus* and *C. albicans* from the mixed biofilm was found to be 4.4 and 3.0, respectively, at 256 μg/mL of β-c-AuNPs. The inhibitory effect of β-c-AuNPs towards single-species biofilms of *S. aureus* and *C. albicans* was found to be higher compared to the mixed biofilms.

### 2.4. Microscopic Examination of Biofilms Treated with β-c-AuNPs

**Figure 7:**
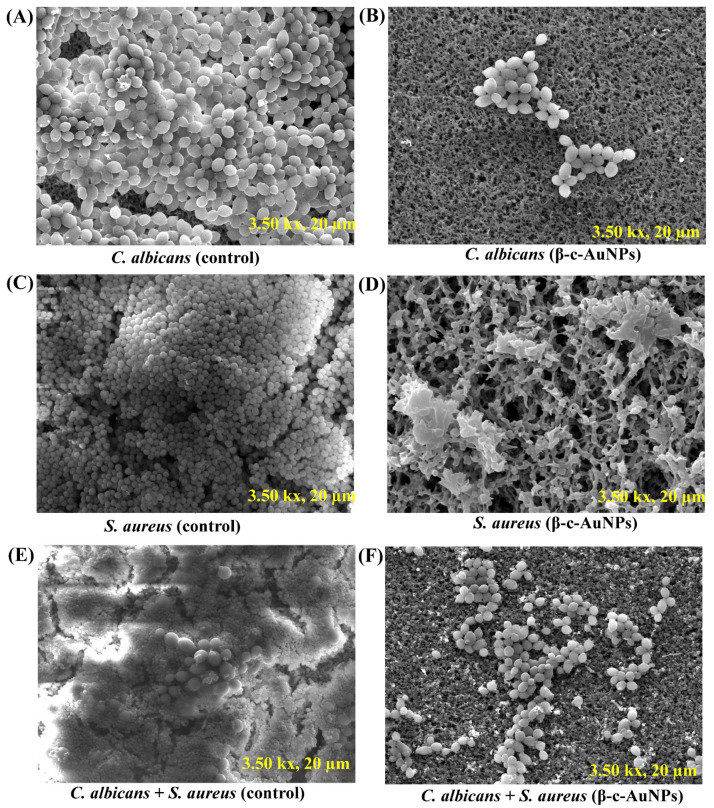
Effect of the subinhibitory concentration of β-c-AuNPs on the initial stage of biofilm formation. (**A**) Biofilms of *C. albicans* (control), (**B**) *C. albicans* biofilm treated with β-c-AuNPs, (**C**) *S. aureus* biofilms (control), (**D**) *S. aureus* biofilms treated with β-c-AuNPs, (**E**) *S. aureus* + *C. albicans* mixed biofilms (control), and (**F**) *S. aureus* + *C. albicans* mixed biofilms treated with β-c-AuNPs.

The SEM analysis further confirmed the inhibitory effects of β-c-AuNPs on single-species as well as mixed biofilms. Our findings here revealed biofilms densely populated with *C. albicans* cells in the control sample (Figure 7A), yet only a few cells on the membrane in the β-c-AuNPs-treated cells (Figure 7B). Similarly, the untreated *S. aureus* biofilms were found to be thicker (Figure 7C) than those treated with β-c-AuNPs (Figure 7D). The surface adherence of both cells was very low in the mixed biofilm of *S. aureus* + *C. albicans* when incubated with β-c-AuNPs, compared to the control mixed biofilms (Figure 7E,F). Based on the SEM analysis, we concluded that the sub-MIC levels of β-c-AuNPs have biofilm inhibitory effects on single- and mixed-species biofilms of bacterial and fungal pathogens.

### 2.5. Effect of β-c-AuNPs towards Mature Biofilms

**Figure 8:**
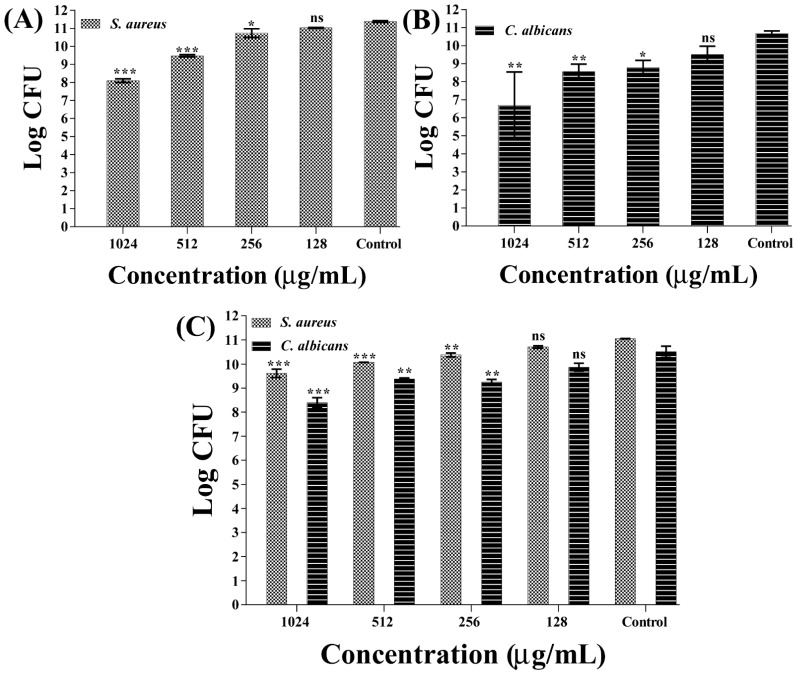
Mature biofilm eradication by β-c-AuNPs. (**A**) CFU of *S. aureus* cells, (**B**) CFU of *C. albicans* cells, and (**C**) CFU of *S. aureus* and *C. albicans* from the mixed mature biofilm. *** *p* < 0.0001, ** *p* < 0.01, and * *p* < 0.05 are statistically significant compared to the control, whereas ns is non-significant.

In addition to the inhibition of initial-stage biofilms, the dispersal of mature single- and mixed-species biofilms was also evaluated using the colony counting method. Different concentrations of β-c-AuNPs were used to test the inhibition of mature biofilms compared to those used in initial-stage biofilm inhibition experiments. The inhibition of mature biofilms was tested using β-c-AuNPs at sub-MIC, MIC, and above MIC values. The inhibition of mature biofilms of *S. aureus* and *C. albicans* was found to be significantly higher at the MIC and above the MIC value of β-c-AuNPs (Figure 8). Accordingly, the reduction in CFU counts of the *S. aureus* biofilm cells at 1024 μg/mL (>MIC) and 512 μg/mL (MIC) were found to be 3.2 log CFU and 1.9 log CFU, respectively (Figure 8A). Similarly, the level of eradication of *C. albicans* mature biofilms at 1024 μg/mL (>MIC) and 512 μg/mL (MIC) was found to be a 3.98 log CFU and 2.11 log CFU reduction of cells, respectively, as compared to the control (Figure 8B). Individual mature biofilms of *S. aureus* and *C. albicans* were eradicated in both cases in a concentration-dependent manner. The CFU counts of the mature mixed biofilm were also significantly reduced at MIC values and above the MIC value of β-c-AuNPs (Figure 8C). The level of reduction of *S. aureus* and *C. albicans* cells in the mixed mature biofilm at the above MIC value (1024 μg/mL) of β-c-AuNPs was found to be 1.43 log CFU and 2.11 log CFU, respectively. Similarly, the reduction of *S. aureus* and *C. albicans* at the MIC value (512 μg/mL) of β-c-AuNPs in the mixed mature biofilm was found to be 0.97 log CFU and 1.12 log CFU, respectively.

## 3. Discussion

The rising number of antimicrobial therapy failures in polymicrobial infections has become a major concern worldwide [43]. The cross-kingdom interaction between pathogenic bacteria and fungal pathogens is a well-studied example of polymicrobial infection [11,44]. In vivo and in vitro studies have shown the existence of synergistic physical and chemical interactions between *S. aureus* and *C. albicans* [10,45,46]. Here, we developed a strategy to control biofilm formation using nanotechnology approaches by using *S. aureus* and *C. albicans* as examples of polymicrobial pathogens. For this purpose, AuNPs were synthesized using a plant-derived compound called β-caryophyllene. Several instrumental techniques were used to characterize the synthesized β-c-AuNPs. The sizes of the β-c-AuNPs were discovered to be relatively small (17.6 ± 1.2 nm), rendering them highly effective in treating microbial pathogens due to their large surface area [47]. Previously, AuNPs synthesized by utilizing a hexane fraction of *O. sanctum* comprising methyl eugenol and β-caryophyllene were likewise found to have a modest size in the range of 1 to 50 nm [28]. Similarly, AgNPs synthesized using β-caryophyllene showed a similar size range of 5 to 100 nm [48].

The synthesized β-c-AuNPs were found to have an irregular shape, similar to those previously reported for AuNPs synthesized using natural products [49]. However, spherical AuNPs synthesized with a hexane fraction containing β-caryophyllene have previously been reported [28]. The high zeta potential of β-c-AuNPs confirms that β-caryophyllene acts as a stabilizing agent, as previously described [28]. Previously, various plant products containing β-caryophyllene have been used to produce a variety of polymeric and metallic nanomaterials, each with a unique size and shape (Table 1). These nanoparticles have been shown to have antimicrobial activities against bacterial and fungal pathogens (Table 1). The β-c-AuNPs synthesized in this study were found to have antimicrobial activity against *S. aureus* and *C. albicans*. Our investigation showed an identical MIC value of β-c-AuNPs against *S. aureus* and *C. albicans*, despite the fact that the MIC values of different AuNPs synthesized using naturally derived chemicals show varying activities against various microbial pathogens [50]. Here, nanoparticles were synthesized using a natural pure compound called β-caryophyllene, which enables the pinpointing of key molecules involved in antimicrobial activity [15]. However, most studies have synthesized nanoparticles using extracts containing β-caryophyllene with a wide spectrum of physiologically active ingredients, making it difficult to specify the components related to nanoparticle formation and biological activity (Table 1).

Polymicrobial biofilms are a major cause of antimicrobial treatment failure, immune system evasion, the development of antimicrobial resistance, and the progression of chronic or persistent infections [51,52]. The mixed biofilms of *S. aureus* and *C. albicans* were exposed to the sub-MIC of β-c-AuNPs to control the initial-stage biofilm formation. The *S. aureus* and *C. albicans* biofilms that developed individually or in mixed forms were inhibited in a concentration-dependent manner. Previous studies have shown similar inhibitory effects of AuNPs synthesized using natural compounds against polymicrobial biofilms of *S. aureus* and *C. albicans* in a concentration-dependent manner [50]. However, drug resistance has become a major challenge in eradicating established mature biofilms [53]. Here, the eradication of mature mixed biofilms of *S. aureus* and *C. albicans* was found to be significant, particularly at and above the MIC of β-c-AuNPs. The eradication effects of β-c-AuNPs on the single-species biofilms of *S. aureus* and *C. albicans* were also higher at and above MIC. The recalcitrant nature of biofilm polymeric substances results in high drug concentrations required for the inhibition of mature biofilms [50,54]. Previous studies have shown that AuNPs synthesized using natural compounds at and above MIC can destroy mature single- or mixed-species biofilms [50,55]. However, because of the enhanced biofilm formation in a synergistic manner in mixed-species biofilms, inhibiting them at the initial stage or eradicating mature biofilms requires a high concentration of drugs [50].

## 4. Materials and Methods

### 4.1. Microbes, Culture Media, and Reagents

The bacterial pathogen *S. aureus* (KCTC 1916) and fungal pathogen *C. albicans* (KCCM 11282) were used as reference strains [42]. The growth media for *S. aureus* and *C. albicans* were tryptic soy broth (TSB) (Difco Laboratory Inc., Detroit, MI, USA) and potato dextrose broth (PDB) with glucose (5%). Growth media containing 50% TSB and 50% PDB with glucose (5%) were used to grow the mixed *S. aureus* and *C. albicans* cell cultures. The culture shock was maintained in 2% glycerol, and culturing was carried out on agar plates (TSA/PDA). Both pathogens were grown at 37 °C. β-caryophyllene (≥98.5%) and gold(III) chloride trihydrate were obtained from Sigma-Aldrich Co. (St. Louis, MO, USA).

### 4.2. Synthesis and Characterization of β-c-AuNPs

The synthesis of β-c-AuNPs was carried out exactly as described previously [50]. In brief, the reaction was carried out in a volume of 200 mL of deionized water by first dissolving 1 mM of HAuCl_4_.3H_2_O (pH 9.0) and stirring at 60 °C. A drop-wise solution of β-caryophyllene (1 mM) was added to the reaction mixture and stirred continuously at 60 °C. During the reaction, the color of the liquid changed from yellow to a deep wine-red. Furthermore, the nanoparticle synthesis was confirmed by measuring the UV-visible absorption spectra using a microplate reader (BioTek, Winooski, VT, USA) with the scanned absorption spectra ranging from 200 to 700 nm. A Fourier transform infrared spectrometer (FTIR, JASCO (FT-4100), Tokyo, Japan) was used to measure the ionic interaction in β-c-AuNPs. The Litesizer 500 was used to determine the size and zeta potential of β-c-AuNPs (Anton Paar, GmbH, Graz, Austria). An XRD (X-Ray Diffractometer, Rigaku (Japan), Ultima IV) was used to determine the crystalline nature of β-c-AuNPs.

### 4.3. Microbroth Dilution for MIC Determination

The antimicrobial activities of β-c-AuNPs were assessed by determining the minimal inhibitory concentration (MIC) against *S. aureus* and *C. albicans*, as recommended by the Clinical and Laboratory Standards Institute (CLSI) [56]. *S. aureus* and *C. albicans* cell cultures (OD_600_ = 0.05) were individually incubated with β-c-AuNPs or β-caryophyllene. The concentration of β-c-AuNPs or β-caryophyllene ranged from 128 to 2048 μg/mL. The treated and untreated cell culture (for the control group) was incubated at 37 °C for 24 h before measuring the OD_600_. As previously reported, tetracycline and fluconazole were used as control drugs to determine the MIC value [42]. The MIC value was calculated based on the absence of visible growth as well as a growth inhibition greater than 90% as measured by the OD_600_. The experiment was carried out in triplicate.

### 4.4. Biofilm Inhibition Assays

The *S. aureus* (OD_600_ = 0.05 in TSB) and *C. albicans* (OD_600_ = 0.05 in PDB with 5 % glucose) cell cultures were incubated separately in a 96-well polystyrene microtiter plate (SPL Life Sciences Co., Ltd. Republic of Korea) containing sub-MIC β-c-AuNPs (ranging from 64 to 256 μg/mL). In the mixed biofilm experiment, 50% *S. aureus* (prepared in TSB) and 50% *C. albicans* cell cultures (prepared in PDB containing 5 % glucose) were added to a microplate containing sub-MIC β-c-AuNPs and incubated for 24 h at 37 °C. The cells that were not treated with β-c-AuNPs were considered the control group. The surface-attached biofilm was counted using the plate counting method described previously [42,50]. After 24 h of incubation, the planktonic cells were removed, and the biofilm cells were rinsed thrice with sterile TSB. The washed cells were scraped off using a sterile pipette tip and resuspended in sterile TSB. The cell culture was serially diluted and plated on an agar plate (TSA for *S. aureus* and PDA for *C. albicans*). The colonies on the agar plates were counted, and the CFU value was computed.

### 4.5. Inhibition of Established Mature Biofilms

The mature biofilm was eradicated using β-c-AuNPs in the same manner as previously described [50]. As mentioned in the biofilm inhibition experiments, the *S. aureus* and *C. albicans* cell cultures were initially allowed to produce mature biofilms independently and in combination. After 24 h of incubation at 37 °C, the planktonic cells were removed and the surface-adhered cells were treated with varying doses of β-c-AuNPs (ranging from 128 to 1024 μg/mL). A control group of untreated cell cultures was used. The microplate was incubated for a further 24 h. The surface-adhered cells were counted using the colony counting method described in the biofilm inhibition assays.

### 4.6. SEM Analysis of Biofilm Architecture

SEM was used to visualize the biofilm cells of *S. aureus* and *C. albicans* treated with β-c-AuNPs, either in mono-species or mixed form [42]. In brief, the cell cultures of single microbial species with an OD_600_ of 0.05 and mixed cultures were placed in a 24-well microplate with a nylon membrane. These cultures were treated with sub-MIC β-c-AuNPs (256 μg/mL) and incubated at 37 °C for 24 h. Formaldehyde (2%) and glutaraldehyde (2.5%) were used to fix the biofilm cells. The fixed cells were washed with phosphate-buffered saline (pH 7.4) and dehydrated in increasing concentrations of ethyl alcohol. The nylon membrane was attached to the SEM stub and visualized using TESCAN (Vega II LSU, Brno, Czech Republic) microscopy at 10 kV voltage and with a magnification of 3.5 kx (20 μm).

### 4.7. Statistical Analysis

GraphPad Prism version 7.0 (GraphPad Software Inc., San Diego, CA) was used to make all the graphs. One-way ANOVA was used for the statistical analysis carried out by one-way ANOVA with Dunnett’s multiple comparisons tests. *p* < 0.0001, *p* < 0.01 and *p* < 0.05 were considered as significant.

## 5. Conclusions

Here, we synthesized β-c-AuNPs to inhibit the polymicrobial biofilms of *S. aureus* and *C. albicans*. The β-c-AuNPs were synthesized using a pure plant-derived compound, β-caryophyllene, with multiple biological roles. Table 1 summarizes the application of several plant materials containing β-caryophyllene in nanoformulations for treating microbiological infections. The synthesized β-c-AuNPs were found to be highly stable with irregular shapes and an average size of 17.6 ± 1.2 nm. β-c-AuNPs were also found to show antimicrobial activities against *S. aureus* and *C. albicans*. The sub-MIC level of β-c-AuNPs inhibited the initial-stage biofilms of *S. aureus* and *C. albicans* as well as the mixed biofilm. These inhibitory effects were concentration-dependent. Furthermore, the mature biofilm eradication of *S. aureus* and *C. albicans* was found to be effective at the MIC and above MIC value of β-c-AuNPs in individual cell cultures and mixed cultures.

Although the synthesized β-c-AuNPs may be biocompatible due to the use of natural compounds, further investigation is still required to examine the toxicity of β-c-AuNPs on different types of animal cells. In future, we intend to focus on elucidating the action mechanism of β-c-AuNPs on the polymicrobial biofilms of *S. aureus* and *C. albicans* by analyzing the biofilm and virulence-related gene expression. Furthermore, the findings of this in vitro study will also be validated in an in vivo system and in host-mimicking media that resemble the host environment.

## Figures and Tables

**Table 1:** Application of various plant materials containing β-caryophyllene in nanoformulation for the treatment of microbiological infections.

Sources	Types	Size	Morphology	Active concentrations	Microbial Pathogens	Mechanism of Antimicrobial Action	References
*Copaifera* sp.	Poly(lactic acid)/polyvinylpyrrolidone nanofibers	920 ± 366 to 1254 ± 600 nm	Continuous and smooth surface	-	*Staphylococcus aureus*	Because polyvinylpyrrolidone is hydrophilic and hygroscopic, it allows for more interaction with the microbial medium.	[29]
*C. multijuga*	Microemulsions	86 ± 15, 83 ± 16 nm	Rounded nanostructure	MIC value of 0.03 to 0.1, 6.45, 1.04 to 6.45, 0.03 to 0.1 mg/mL	*S. aureus* *Escherichia coli* *Pseudomonas aeruginosa* *Cryptococcus neoformans*	Increased cell penetration due to the high surfactant content.	[30]
*C. guaianensis* *C. officinalis*	Nanoemulsions	192.36 ± 1.30 nm 211.53 ± 1.65 nm	-	MIC value of 0.39 to 6.25%	*Paenibacillus* species	-	[31]
*Phlomoides labiosa*	Nanoliposomes	480, 520, 643 nm	Spherical	-	*E. coli* *S. epidermidis* *S. aureus*	-	[32]
Clove oil	Chitosan nanoparticles Gelatin electrospun nanofibers	154.9 ± 3.0 to 236.3 ± 2.6 nm 125 to 375 nm	Smooth morphology Thin and smooth	MBC value of 3, 6, 9 mg/mL	*E. coli* O157:H7	-	[33]
*Lantana camara* L. leaves	Silver nanoparticles	425 nm	Spherical	-	*S. aureus* *E. coli* *P. aeruginosa*	The polymer subunits of the cell membrane and bacterial protein synthesis were both disrupted.	[34]
*Thymus vulgaris*	Nanoemulsion	52 nm	Spherical	-	*Salmonella Typhimurium* *E. coli* *S. aureus* *Bacillus cereus* *Listeria monocytogenes* *Aspergillus niger* *A. flavus*	The outer membrane was damaged, resulting in the leakage of lipopolysaccharides.	[35]
*Tetragastris catuaba* *Pure compound*	Nanoemulsions	40.7 ± 0.07 nm 41.7 ± 0.41 nm	Small droplets Dispersed spheres	MIC value of 0.5 to 8 mg/mL	*Enterococcus faecalis* *L. monocytogenes*	-	[36]
Pure compound	Halloysite nanotubes	~900 to ~1100 nm	Flat ribbon-like morphology	-	*B. subtilis* *E. coli*	Changed the permeability of the cell membrane, resulting in cell wall damage and intracellular substance leakage.	[37]
Pure compound	Lipidic nanocapsules	66 ± 4 nm	-	MIC value of 0.62 ± 0.01 mg/mL	*Acinetobacter baumannii*	This resulted in the leakage of intracellular components	[38]

## Data Availability

Not applicable.

## References

[1] Peters B.M., Jabra-Rizk M.A., O’May G.A., Costerton J.W., Shirtliff M.E. (2012). Polymicrobial interactions: Impact on pathogenesis and human disease. Clin. Microbiol. Rev..

[2] Vega N.M., Allison K.R., Samuels A.N., Klempner M.S., Collins J.J. (2013). *Salmonella typhimurium* intercepts Escherichia coli signaling to enhance antibiotic tolerance. Proc. Natl. Acad. Sci. USA.

[3] Korgaonkar A., Trivedi U., Rumbaugh K.P., Whiteley M. (2013). Community surveillance enhances *Pseudomonas aeruginosa* virulence during polymicrobial infection. Proc. Natl. Acad. Sci. USA.

[4] Hu Y., Niu Y., Ye X., Zhu C., Tong T., Zhou Y., Zhou X., Cheng L., Ren B. (2021). *Staphylococcus aureus* Synergized with *Candida albicans* to Increase the Pathogenesis and Drug Resistance in Cutaneous Abscess and Peritonitis Murine Models. Pathogens.

[5] Schlecht L.M., Peters B.M., Krom B.P., Freiberg J.A., Hänsch G.M., Filler S.G., Jabra-Rizk M.A., Shirtliff M.E. (2015). Systemic *Staphylococcus aureus* infection mediated by *Candida albicans* hyphal invasion of mucosal tissue. Microbiology.

[6] Khan F., Jeong G.-J., Javaid A., Pham D.T.N., Tabassum N., Kim Y.-M. (2022). Surface adherence and vacuolar internalization of bacterial pathogens to the Candida spp. cells: Mechanism of persistence and propagation. J. Adv. Res..

[7] Klotz S.A., Chasin B.S., Powell B., Gaur N.K., Lipke P.N. (2007). Polymicrobial bloodstream infections involving *Candida* species: Analysis of patients and review of the literature. Diagn. Microbiol. Infect. Dis..

[8] O’Donnell L.E., Millhouse E., Sherry L., Kean R., Malcolm J., Nile C.J., Ramage G. (2015). Polymicrobial *Candida* biofilms: Friends and foe in the oral cavity. FEMS Yeast Res..

[9] Carlson E. (1983). Effect of strain of *Staphylococcus aureus* on synergism with *Candida albicans* resulting in mouse mortality and morbidity. Infect. Immun..

[10] Carlson E. (1982). Synergistic effect of *Candida albicans* and *Staphylococcus aureus* on mouse mortality. Infect. Immun..

[11] Khan F., Bamunuarachchi N.I., Pham D.T.N., Tabassum N., Khan M.S.A., Kim Y.M. (2021). Mixed biofilms of pathogenic *Candida*-bacteria: Regulation mechanisms and treatment strategies. Crit. Rev. Microbiol..

[12] Kong E.F., Tsui C., Kucharíková S., Andes D., Van Dijck P., Jabra-Rizk M.A. (2016). Commensal Protection of *Staphylococcus aureus* against Antimicrobials by *Candida albicans* Biofilm Matrix. mBio.

[13] De Oliveira D.M.P., Forde B.M., Kidd T.J., Harris P.N.A., Schembri M.A., Beatson S.A., Paterson D.L., Walker M.J. (2020). Antimicrobial Resistance in ESKAPE Pathogens. Clin. Microbiol. Rev..

[14] Fisher M.C., Denning D.W. (2023). The WHO fungal priority pathogens list as a game-changer. Nat. Rev. Microbiol..

[15] Khan F., Jeong G.J., Singh P., Tabassum N., Mijakovic I., Kim Y.M. (2022). Retrospective analysis of the key molecules involved in the green synthesis of nanoparticles. Nanoscale.

[16] Kang M.-G., Khan F., Jo D.-M., Oh D., Tabassum N., Kim Y.-M. (2022). Antibiofilm and Antivirulence Activities of Gold and Zinc Oxide Nanoparticles Synthesized from Kimchi-Isolated *Leuconostoc* sp. Strain C2. Antibiotics.

[17] Jeong G.-J., Khan S., Tabassum N., Khan F., Kim Y.-M. (2022). Marine-bioinspired nanoparticles as potential drugs for multiple Biological Roles. Mar. Drugs.

[18] Alavi M., Kowalski R., Capasso R., Douglas Melo Coutinho H., Rose Alencar de Menezes I. (2022). Various novel strategies for functionalization of gold and silver nanoparticles to hinder drug-resistant bacteria and cancer cells. Micro. Nano. Bio. Aspects.

[19] Alavi M., Hamblin M.R., Kennedy J.F. (2022). Antimicrobial applications of lichens: Secondary metabolites and green synthesis of silver nanoparticles: A review. Nano. Micro. Biosystems.

[20] Xu Z., Zhang C., Wang X., Liu D. (2021). Release strategies of silver ions from materials for bacterial killing. ACS Appl. Bio Mater..

[21] Gu X., Xu Z., Gu L., Xu H., Han F., Chen B., Pan X. (2021). Preparation and antibacterial properties of gold nanoparticles: A review. Environ. Chem. Lett..

[22] Huang M., Sanchez-Moreiras A.M., Abel C., Sohrabi R., Lee S., Gershenzon J., Tholl D. (2012). The major volatile organic compound emitted from *Arabidopsis thaliana* flowers, the sesquiterpene (E)-β-caryophyllene, is a defense against a bacterial pathogen. New Phytol..

[23] Takemoto Y., Kishi C., Sugiura Y., Yoshioka Y., Matsumura S., Moriyama T., Zaima N. (2021). Distribution of inhaled volatile β-caryophyllene and dynamic changes of liver metabolites in mice. Sci. Rep..

[24] Fidyt K., Fiedorowicz A., Strządała L., Szumny A. (2016). β-caryophyllene and β-caryophyllene oxide—Natural compounds of anticancer and analgesic properties. Cancer Med..

[25] Moo C.-L., Yang S.-K., Osman M.-A., Yuswan M.H., Loh J.-Y., Lim W.-M., Swee-Hua-Erin L., Lai K.-S. (2020). Antibacterial Activity and Mode of Action of β-caryophyllene on. Pol. J. Microbiol..

[26] Jung D.H., Park M.H., Kim C.J., Lee J.Y., Keum C.Y., Kim I.S., Yun C.-H., Kim S.-k., Kim W.H., Lee Y.C. (2020). Effect of β-caryophyllene from Cloves Extract on *Helicobacter pylori* Eradication in Mouse Model. Nutrients.

[27] Purkait S., Bhattacharya A., Bag A., Chattopadhyay R. (2020). Evaluation of antibiofilm efficacy of essential oil components β-caryophyllene, cinnamaldehyde and eugenol alone and in combination against biofilm formation and preformed biofilms of *Listeria monocytogenes* and *Salmonella typhimurium*. Lett. Appl. Microbiol..

[28] Lee S.Y., Krishnamurthy S., Cho C.-W., Yun Y.-S. (2016). Biosynthesis of Gold Nanoparticles Using *Ocimum sanctum* Extracts by Solvents with Different Polarity. ACS Sustain. Chem. Eng..

[29] Bonan R.F., Bonan P.R., Batista A.U., Sampaio F.C., Albuquerque A.J., Moraes M.C., Mattoso L.H., Glenn G.M., Medeiros E.S., Oliveira J.E. (2015). *In vitro* antimicrobial activity of solution blow spun poly (lactic acid)/polyvinylpyrrolidone nanofibers loaded with Copaiba (*Copaifera* sp.) oil. Mater. Sci. Eng. C.

[30] de Oliveira Neves J.K., Apolinário A.C., Saraiva K.L.A., da Silva D.T.C., Reis M.Y.d.F.A., de Lima Damasceno B.P.G., Pessoa A., Galvão M.A.M., Soares L.A.L., da Veiga Júnior V.F. (2018). Microemulsions containing *Copaifera multijuga* Hayne oil-resin: Challenges to achieve an efficient system for β-caryophyllene delivery. Ind. Crops Prod..

[31] de Almeida Vaucher R., Giongo J.L., Bolzan L.P., Côrrea M.S., Fausto V.P., dos Santos Alves C.F., Lopes L.Q.S., Boligon A.A., Athayde M.L., Moreira A.P. (2015). Antimicrobial activity of nanostructured Amazonian oils against *Paenibacillus* species and their toxicity on larvae and adult worker bees. J. Asia-Pac. Entomol..

[32] Mohammadi S., Valizadeh H., Khaleseh F., Bastani S., Delazar A., Asgharian P. (2022). Biological activities of extract-loaded nanocarriers: A comparison of aerial part, seed, and rhizome of *Phlomoides labiosa*. Eur. J. Integr. Med..

[33] Cui H., Bai M., Rashed M.M., Lin L. (2018). The antibacterial activity of clove oil/chitosan nanoparticles embedded gelatin nanofibers against *Escherichia coli* O157: H7 biofilms on cucumber. Int. J. Food Microbiol..

[34] Shriniwas P.P., Subhash T.K. (2017). Antioxidant, antibacterial and cytotoxic potential of silver nanoparticles synthesized using terpenes rich extract of *Lantana camara* L. leaves. Biochem. Biophys. Rep..

[35] El-Sayed S.M., El-Sayed H.S. (2021). Antimicrobial nanoemulsion formulation based on thyme (*Thymus vulgaris*) essential oil for UF labneh preservation. J. Mater. Res. Technol..

[36] da Silva R.C.S., de Souza Arruda I.R., Malafaia C.B., de Moraes M.M., Beck T.S., da Camara C.A.G., da Silva N.H., da Silva M.V., dos Santos Correia M.T., Frizzo C.P. (2022). Synthesis, characterization and antibiofilm/antimicrobial activity of nanoemulsions containing *Tetragastris catuaba* (Burseraceae) essential oil against disease-causing pathogens. J. Drug Deliv. Sci. Technol..

[37] Ullah A., Sun L., Wang F.-f., Nawaz H., Yamashita K., Cai Y., Anwar F., Khan M.Q., Mayakrishnan G., Kim I.S. (2023). Eco-friendly bioactive β-caryophyllene/halloysite nanotubes loaded nanofibrous sheets for active food packaging. Food Packag. Shelf Life.

[38] Montagu A., Saulnier P., Cassisa V., Rossines E., Eveillard M., Joly-Guillou M.-L. (2014). Aromatic and Terpenic Compounds Loaded in Lipidic Nanocapsules: Activity against Multi-drug Resistant *Acinetobacter baumannii* Assessed *in vitro* and in a Murine Model of Sepsis. J. Nanomed. Nanotechnol..

[39] Altaf M., Zeyad M.T., Hashmi M.A., Manoharadas S., Hussain S.A., Abuhasil M.S.A., Almuzaini M.A.M. (2021). Effective inhibition and eradication of pathogenic biofilms by titanium dioxide nanoparticles synthesized using *Carum copticum* extract. RSC Adv..

[40] Yadav T.C., Gupta P., Saini S., Mohiyuddin S., Pruthi V., Prasad R. (2022). Plausible Mechanistic Insights in Biofilm Eradication Potential against *Candida* spp. Using In Situ-Synthesized Tyrosol-Functionalized Chitosan Gold Nanoparticles as a Versatile Antifouling Coating on Implant Surfaces. ACS Omega.

[41] Villa-García L.D., Márquez-Preciado R., Ortiz-Magdaleno M., Patrón-Soberano O.A., Álvarez-Pérez M.A., Pozos-Guillén A., Sánchez-Vargas L.O. (2021). Antimicrobial effect of gold nanoparticles in the formation of the *Staphylococcus aureus* biofilm on a polyethylene surface. Braz. J. Microbiol..

[42] Khan F., Oh D., Chandika P., Jo D.M., Bamunarachchi N.I., Jung W.K., Kim Y.M. (2022). Inhibitory activities of phloroglucinol-chitosan nanoparticles on mono- and dual-species biofilms of *Candida albicans* and bacteria. Colloids Surf. B Biointerfaces.

[43] O’Brien T.J., Figueroa W., Welch M. (2022). Decreased efficacy of antimicrobial agents in a polymicrobial environment. ISME J..

[44] Shirtliff M.E., Peters B.M., Jabra-Rizk M.A. (2009). Cross-kingdom interactions: *Candida albicans* and bacteria. FEMS Microbiol. Lett..

[45] Todd O.A., Fidel P.L., Harro J.M., Hilliard J.J., Tkaczyk C., Sellman B.R., Noverr M.C., Peters B.M. (2019). *Candida albicans* Augments *Staphylococcus aureus* Virulence by Engaging the Staphylococcal agr Quorum Sensing System. mBio.

[46] Van Dyck K., Viela F., Mathelié-Guinlet M., Demuyser L., Hauben E., Jabra-Rizk M.A., Vande Velde G., Dufrêne Y.F., Krom B.P., Van Dijck P. (2021). Adhesion of *Staphylococcus aureus* to *Candida albicans* during Co-Infection Promotes Bacterial Dissemination Through the Host Immune Response. Front. Cell. Infect. Microbiol..

[47] Alsamhary K., Al-Enazi N., Alshehri W.A., Ameen F. (2020). Gold nanoparticles synthesised by flavonoid tricetin as a potential antibacterial nanomedicine to treat respiratory infections causing opportunistic bacterial pathogens. Microb. Pathog..

[48] Kamaraj C., Balasubramani G., Siva C., Raja M., Balasubramanian V., Raja R.K., Tamilselvan S., Benelli G., Perumal P. (2017). Ag Nanoparticles Synthesized Using β-Caryophyllene Isolated from Murraya koenigii: Antimalarial (*Plasmodium falciparum* 3D7) and Anticancer Activity (A549 and HeLa Cell Lines). J. Clust. Sci..

[49] Mariychuk R., Fejer J., Linnik R.P., Grishchenko L.M., Lisnyak V.V. (2023). Green synthesis and photoluminescence properties of gold nanoparticles with irregular shapes. Mol. Cryst. Liq. Cryst..

[50] Tabassum N., Khan F., Kang M.-G., Jo D.-M., Cho K.-J., Kim Y.-M. (2023). Inhibition of Polymicrobial Biofilms of *Candida albicans*-*Staphylococcus aureus*/*Streptococcus mutans* by Fucoidan-Gold Nanoparticles. Mar. Drugs.

[51] Gabrilska R.A., Rumbaugh K.P. (2015). Biofilm models of polymicrobial infection. Future Microbiol..

[52] Anju V.T., Busi S., Imchen M., Kumavath R., Mohan M.S., Salim S.A., Subhaswaraj P., Dyavaiah M. (2022). Polymicrobial Infections and Biofilms: Clinical Significance and Eradication Strategies. Antibiotics.

[53] Singh S., Datta S., Narayanan K.B., Rajnish K.N. (2021). Bacterial exo-polysaccharides in biofilms: Role in antimicrobial resistance and treatments. J. Genet. Eng. Biotechnol..

[54] Xia W., Li N., Shan H., Lin Y., Yin F., Yu X., Zhou Z. (2021). Gallium porphyrin and gallium nitrate reduce the high vancomycin tolerance of MRSA biofilms by promoting extracellular DNA-dependent biofilm dispersion. ACS Infect. Dis..

[55] Shilpha J., Meyappan V., Sakthivel N. (2022). Bioinspired synthesis of gold nanoparticles from *Hemidesmus indicus* L. root extract and their antibiofilm efficacy against *Pseudomonas aeruginosa*. Process Biochem..

[56] Wayne P. (2011). Clinical and Laboratory Standards Institute. Performance Standards for Antimicrobial Susceptibility Testing. Twenty-First Informational Supplement. Document M100-S21. https://www.aeciherj.org.br/publicacoes/clsi.pdf.

